# American Veterinary Medical Association

**Published:** 1901-04

**Authors:** S. Stewart

**Affiliations:** Secretary; Kansas City, Missouri


### AMERICAN VETERINARY MEDICAL ASSOCIATION.

Editor Journal Comparative Medicine:

Dear Doctor: The members of the A. V. M. A. have doubtless noted with much interest the decision of the executive committee to hold the next annual meeting in Atlantic City; also, the announcement that a committee of prominent members had been appointed to serve as committee on local arrangements, and that this committee was already preparing to make the coming meeting very attractive.

It is hoped by the secretary that during the winter months each member has been saying to himself that he would attend this meeting, and that he would be pleased to have a share in making it one of power and uplift for the Association and the profession in general; that he would be pleased to contribute a paper for the programme, and had, in fact, collected some material for such a paper. The secretary also hopes that the members will not await a personal invitation from him to contribute such paper, but will feel that it is his privilege and his duty to do his part toward making the meeting a success, and that he will at once notify the secretary that he desires to offer a paper and will state the title of the same. The following members have signified their intention to offer papers, to wit: Dr. D. F. Yonkerman, Kalamazoo, Mich.; Dr. George W. Dunphy, Quincy, Mich.; Dr. William McEachran, Windsor, Ont.; Dr. C. A. Cary, Auburn, Ala.; and Dr. W. H. Dalrymple, Baton Rouge, La. There will be abundant time for the presentation and proper discussion of several times this number, and it is hoped that the privilege of presenting a paper for the Association will be so highly prized that offers of papers will be sent in abundance.

The members residing west of Buffalo are saying that they will arrange to attend the Exposition at Buffalo en route to or from the meeting, this plan affording a very greatly reduced transportation to Buffalo, and from Buffalo to Atlantic City and return. Never before have the veterinarians of the western and central part of the Continent seemed to take such early and general interest in the coming meeting of the Association as they have done this year, if the secretary's correspondence is any guide, and it behooves the veterinarians of the East to awake to this opportunity to meet their Western confrères and to show them how good and how pleasant it is to live on the Atlantic Coast.

The report of the proceedings of the Detroit meeting was printed and distributed about December 1, 1900, and a copy was sent to each member, both old and new. The numerous inquiries received as to whether or not the proceedings have been published leads me to suspect that in numerous instances the report was not delivered. If any of the members who read this failed to receive a copy they should notify me at once in order that the missing copy may be looked up and delivered.

Yours very truly,

S. Stewart,

Kansas City, Missouri. Secretary.

---

Dr. T. Earle Budd was among the Essex Troop at Washington, D. C., during the inaugural ceremonies.

Drs. Leonard Pearson and M. E. Conard, of Philadelphia, spent many days at Harrisburg during the month of March looking after legislation of interest to the veterinary profession and the live-stock industries of the State.

